# The lignin fluorescence at the base of the petals guiding the pollinators to the pollination chamber in *Artabotrys hainanensis*

**DOI:** 10.1186/s12870-026-08769-3

**Published:** 2026-04-23

**Authors:** Bingxin Li, Xiu Liu, Yanli Lin, Fengxia Xu

**Affiliations:** 1 South China Botanical Garden, Chinese Academy of Sciences, Guangzhou, 510650 China; 2 National Park Research Center, School of Economics and Management, Sanming University, Sanming, 365004 China; 3 Fujian Provincial Engineering research Center of the Development and Utilization of Medicinal Plants, Sanming University, Sanming, 365004 China; 4 College of Life Sciences, University of Chinese Academy of Sciences, Beijing, 100000 China

**Keywords:** Annonaceae, *Artabotrys hainanensis*, Fluorescence, Lignin, Pollination chamber

## Abstract

In *Artabotrys hainanensis*, the pollination chamber aperture of the flowers at the blooming stage is narrow, and only exposed in the pistillate phase. It is worth studying how the pollinating insects enter the pollination chambers accurately through the narrow aperture. During flowering, the dynamics of the opening and closing of the pollination chambers ensure that sufficient pollen is carried by pollinating beetles (Carpophilinae) facilitating successful pollination. *A. hainanensis* exhibits fluorescence at the base of the inner and outer whorls of petals, which marks the entrance to the pollination chamber, acting a role in pollinator attraction. The lignin content gradually accumulated as the trichomes in pollination chamber areas increase in density and volume during flower development, and the lignin fluorescent signals at the pollination chamber areas reach their brightest at the blooming stage. The green fluorescence emitted by lignin under visible light could attract beetles, which have visual receptors that are sensitive to green light. Therefore, the lignin fluorescence produced by trichomes that located at the aperture of the pollination chamber serves as a visual signal that guides the pollinating insects through the narrow pollination aperture and into the flower pollination chamber at anthesis. Further studies are required to determine if lignin-associated fluorescence signals occur in other species of the Annonaceae and if they represent a common trait within the family.

## Background

 Fluorescence as a common phenomenon in the flowers, leaves, stalks and roots of plant organisms has some functions, such as biocommunication roles, ultraviolet defense, etc [1]. The most common fluorescent substances in plants are lignin and chlorophyll. Additionally, there are numerous other fluorescent molecules in plants, including pigments, tannins, flavonoids, phenolic, etc [1, 2]. Because flowers are important for plant reproduction, the fluorescence phenomenon in flowers has received much attention. The fluorescence appears in different structures of flowers, such as the nectary [3], stigma [4], ovary [4], pollen [5, 6], and petals [7, 8]. It plays an important role in the biocommunication between flowering plants and pollinators [1, 7]. To date, reports on fluorescent compounds that attract pollinators in flowers are limited. The blue fluorescence emitted by the hydroxycinnamoyl derivatives in pollen and anthers serves as a visual cue to attract honeybees under sunlight [9]. According to Gandía-Herrero et al. [7, 8], the betaxanthins in the petals of *Portulaca grandiflora* and *Mirabilis jalapa* produce a green fluorescence that may serve as a visual signal to attract pollinators. Also, the ferulic acid in petals, stamens and pollen emitted blue fluorescence in grasses plays a potential role in insect attraction [6]. However, only two fluorescent compounds, ferulic acid and betaxanthins, have been reported in petals, which may serve as a visual cue to attract pollinators [6–8].

Lignin, a complex polyphenolic polymer synthesized in plants, is an important component of the secondary cell wall and widely distributed in plant stems and roots where it plays key physiological functions in structural support, water transport, and disease defense [10]. As a natural fluorescent substance, lignin contains diverse fluorophores with a wide range of emission spectra, emitting blue, green, and red fluorescence under UV and visible light excitation [11, 12]. The fluorescent properties of lignin have been extensively used in cellular imaging and in studies of the structural characteristics of plant tissues, including investigating the cellular structure and cell types of xylem and vascular bundles, as well as the structure of wood-based products [10, 12–14]. Regarding the distribution of lignin in the flower structure, the reports in this area remain scarce. To date, the function of lignin fluorescence in flowers seems have never been recognized.

Annonaceae are a diverse family of flowering trees and climbers widespread in wet lowland and lower montane forests throughout the tropics. Annonaceae flowers possess whorls of distinct sepals and petals. The two petal whorls are generally morphologically distinct, with the inner petals often convergent or connivent to form a partially enclosed pollination chamber [15]. The pollination chamber provides a place for insects to feed and mate [16–18]. In some cases, it also plays as a trap [19, 20]. *Artabotrys hainanensis*, a species of Annonaceae, exhibits a tightly enclosed pollination chamber with an elaborate rim between the inner petal blade and claw. The pollination chamber aperture of the flowers at the blooming stage is narrow and its opening time is only about 8 h in *A*. *hainanensis*. We aimed to explore how insects accurately locate and enter the pollination chamber in *A*. *hainanensis*, by monitoring the floral phenology and pollinator circadian rhythms, analyzing the fluorescence of petal under blue light, comparing the morphology and structure of trichomes in petal, and the lignin content of the petals during flower development stages.

## Materials and methods

*Artabotrys hainanensis* R. E. Fries is a climbing shrub that inhabits subtropical forests in southeastern China. *Artabotrys hainanensis* was identified by Prof. Xueliang Hou. Voucher specimens have been deposited in the publicly available herbarium of the South China Botanical Garden, Chinese Academy of Sciences (IBSC:0077396). Field observations of *A. hainanensis* were conducted in the South China National Botanical Garden, Guangdong Province, China (113.373^◦^N, 23.188^◦^E).

### Floral phenology

Phenological studies were conducted over two successive flowering seasons (2021–2022) to identify the timing and duration of floral anthesis. A total of 50 flower buds were tagged and monitored every day and hourly for bud development and anthesis, respectively. The onset and duration of stigmatic receptivity, presence or absence of stigmatic exudate and fragrance, and color changes in the petal and stigmas were recorded throughout the pistillate phase. The presence of stigmatic exudate was observed to be correlated with stigmatic receptivity and was accordingly used as supporting evidence for determining the onset of the pistillate phase [21]. Stigmatic receptivity was confirmed by immersing the stigmas in 3% hydrogen peroxide (H_2_O_2_) solution and observing effervescence, indicative of peroxidase activity [22]. The staminate phase was recognized by anther dehiscence, and associated color changes recorded throughout the staminate phase. In addition, the presence of an interim phase of asexual functioning between the end of the pistillate phase and the beginning of the staminate phase was recorded.

### Petal fluorescence survey

The whole flower and inner and outer petals of *A. hainanensis* at different developmental stages were observed and photographed under white light and blue light in a fluorescence stereomicroscope (LEICA 205FA), respectively, with the excitation wavelength of blue light at 450–490 nm. For each developmental stage, at least five biological replicates were analyzed. The average fluorescence intensity in the pollination chamber areas were measured using ImageJ software, and the resulting data were analyzed in SPSS and plotted in Excel.

### Epidermal hair morphological observation

The petal of buds and mature flowers of *A. hainanensis* from differentiation up to anthesis were collected from September 2021 to November 2022, and separately immediate fixed in prepared 2.5% glutaraldehyde (4℃) or in formalin acetic alcohol (FAA (v/v): 70% alcohol, formaldehyde, and glacial acetic acid at a ratio of 18:1:1) for more than 24 h to preserve. After fixation in FAA, petal materials from different developmental stages were dehydrated and embedded in paraffin. Serial sections of 8 μm thick were prepared using automatic rotary microtome (Thermo Scientific HM355S) and stained with Hematoxylin-Eosin. The other part of the paraffin section without staining was directly placed under fluorescence stereomicroscope and observed by blue light irradiation.

The fresh petal materials of *A. hainanensis* at different developmental stage and in different parts of the petal that fixed in 2.5% glutaraldehyde, followed by dehydration in a graded water-ethanol series, and dried with a CO_2_ critical-point dryer. After being sputter-coated with gold, the dried petals were examined with a JSM-6360LV (JEOL, Tokyo, Japan) scanning electron microscope operated at 25 kV.

### Fluorescent compound identification

Fresh petal segments (1 cm × 1 cm) from both the pollination chamber and non-pollination chamber areas were dissected and immersed in a dissociative solution (30% hydrogen peroxide: acetic acid (v/v) = 1:1), and incubated in a thermostatic water bath at 60℃ for 2 h. After the petal tissues and epidermal cells were separated, the dissociated materials were removed, and the broken tissues and cells were gently swept away with a brush and rinsed in water. The upper epidermis of different parts of the petals were placed on slides dripping with water, sealed with glycerol. Another dissociated petal epidermis was placed on a slide and a drop of 2% phloroglucinol ethanol solution (2 g of phloroglucinol dissolved in 100 mL of 95% ethanol) on the sample stained for 2 min, and then an equal amount of 25% HCl was dripped in to visualize the coloration for 2 min, and then the excess of the staining solution was sucked off, covered with a coverslip. All the above sections were observed and photographed using a light microscope (LEICA DM5500 B).

Fresh petal epidermal materials of *A. hainanensis* flowers at different developmental stages were collected and assayed for lignin content using the acetyl bromide method, with reference to Fang et al. [23]. The lignin content was measured in the pollination chamber areas of inner and outer whorl petals as well as in the non-pollination chamber areas, respectively. A standard curve was generated using purified lignin, with glacial acetic acid serving as a blank control. The UV absorption values of the samples were measured at 280 nm, with three independent biological replicates performed.

### Floral visitors and visiting behavior analysis

We observed floral visitors to 50 flowers of *A. hainanensis* at the South China National Botanical Garden in 2021–2022. Flowers were observed throughout anthesis at 1 h intervals between 9:00 a.m. and 9:00 p.m. each day to record the arrival and departure patterns of the floral visitors. The following criteria were used to determine whether floral visitors were effective pollinators: (1) relative visitation rates; (2) the coincidence of visits and the duration of pistillate or staminate phases in the flowers; (3) the attachment of *A. hainanensis* pollen grains to the floral visitors, determined using LM (light microscopy) and/or SEM (scanning electron microscopy); and (4) evidence of movement of floral visitors between flowers of different anthesis phases [24]. The floral visitor was captured into plastic centrifuge tube and after checking for attached pollen grains, preserved in 70% alcohol for further identification [24].

Flowers of *A. hainanensis* in the pistillate phase were collected. Pollinators hidden within the pollination chambers of flowers in the interim and staminate phases were captured in the morning and transported to the laboratory. The collected flowers in the pistillate phase were divided into two groups, A and B, in which the flowers of group A were painted black at the pollination chamber areas to block the fluorescent of the petals, and the flowers of group B were without any treatment, and three flowers of each group were placed in two glass vials, and four pollinators were carefully put into each glass vial, the caps of the vials were then sealed to prevent the beetles from flying away. The behavior of insects visiting flowers (e.g., visitation time, approach behavior toward the pollination chamber, visitation frequency, etc.) was observed and recorded under both natural light and blue light. Each observation session lasted 4–5 h, and the experiment was replicated five times, encompassing a total of 30 flowers.

## Results

### Floral phenology

**Fig. 1:**
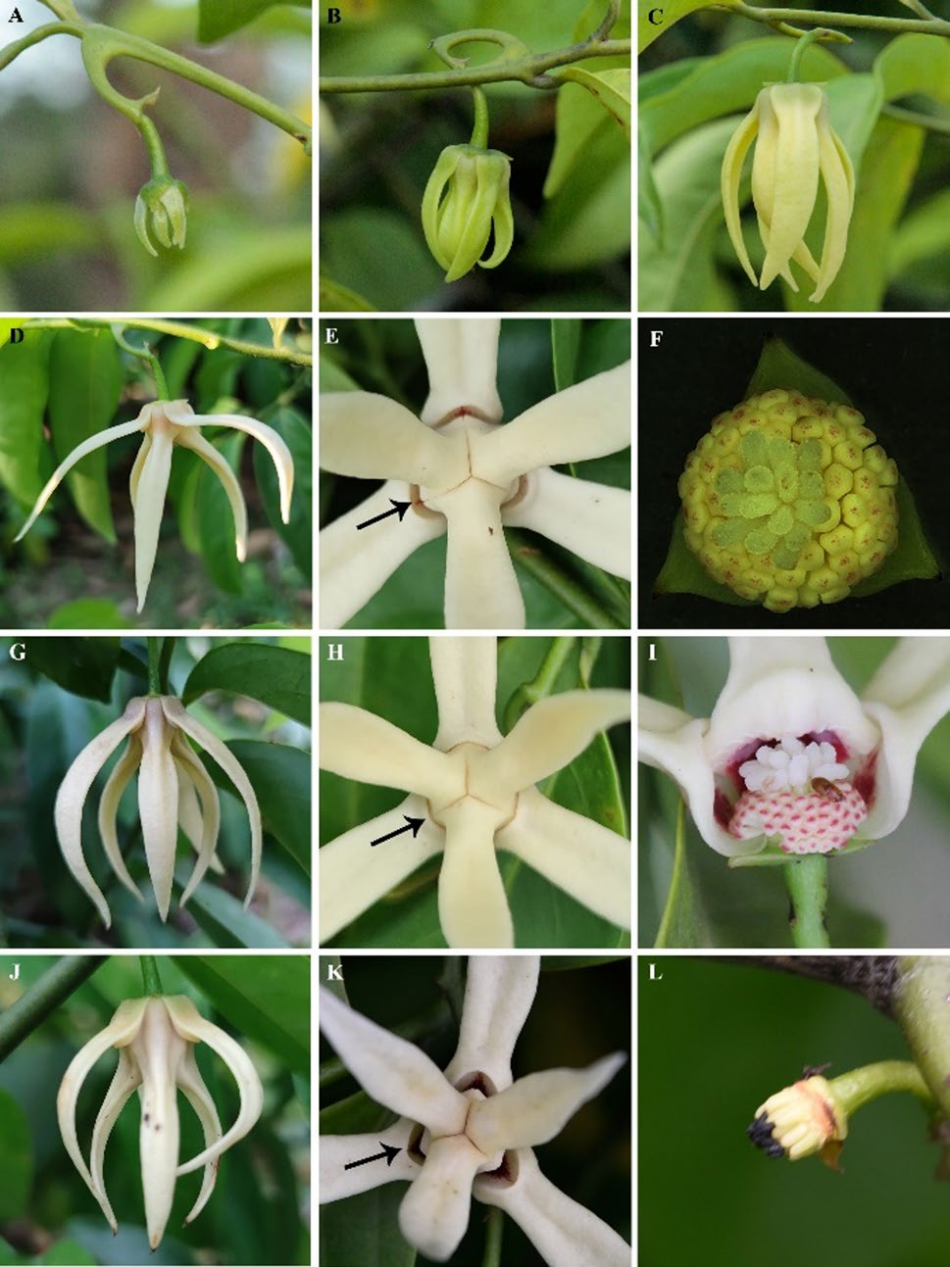
Flowers of *Artabotrys hainanensis* at different phenological stages. **A**: Early bud stage; **B**: Petal elongation stage; **C**: Pollination chamber formation stage; **D**-**F**: Pistillate phase, outer petals extend outward, exposing the pollination chamber holes (arrows); **G**-**I**: Interim phase, outer wheel petals pendulous, arrows indicate pollinating hole closed; **J**-**L**: Staminate phase, outer petals extend outward, exposing the pollination chamber holes (arrows); **L**: End of staminate phase, petals and stamens detached from receptacle

**Fig. 2:**
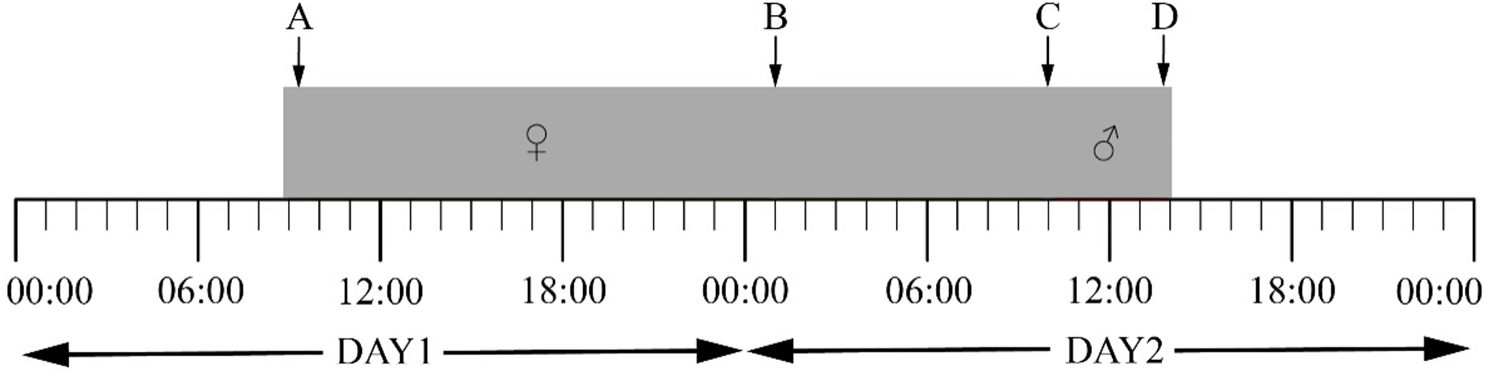
Timing of anthesis of *Artabotrys hainanensis*. **A**: Initiation of pistillate phase, rise of outer petals and exposing the basal apertures; **B**: Initiation of interim phase, close of outer petals and blocking the basal apertures; **C**: Initiation of staminate stage; **D**: End of staminate stage, abscission of petals and stamens, and departure of pollinators

The flowers of *A. hainanensis* have two whorl petals, with three petals per whorl, the base of each petal is concave and converges to form a closed pollination chamber that surrounds the reproductive organs (Fig. 1). Flowers are hermaphroditic and protogynous, with anthesis lasting over two days (Fig. 2). Duration from the early bud stage to the end of flowering lasts about 32 days (Fig. 1), and six different stages of floral development are apparent as follows:

- Stage I: early bud (ca. 15d). Flower buds are green, petal length approximate 0.5cm, with initially the larger sepals enclosing the petals, which beginning to separate after 2 or 3 days, exposing the outer whorl of petals, with the apical portion of the inner whorl of petals are visible (Fig. 1A).
- Stage II: petal elongation (7-8d). Both whorls of petals remain green, petal length approximate 1.5cm. At this stage the petals elongate rapidly, and as the petals progress the base of inner whorl petals begin to concave and tightly compress against each other forming the prototype of the pollination chamber, while this curvature is not obvious on the outer petals. The flowers gradually become pendulous with increasing weight (Fig. 1B).
- Stage III: pollination chamber formation (ca. 8d). The petals of the inner and outer whorls continue to enlarge and gradually turn yellow, petal length approximate 3-4cm, with the bases of the inner and outer whorl petals become constricted and inwardly curved and the close-type pollination chamber fully developed. The pollination chambers have three apertures located between the basal edges of adjacent inner whorl petals and are sealed by the outer whorl petals (Fig. 1C).
- Stage IV: pistillate phase (ca. 8h). The pistillate stage commenced at approximately 9:00 a.m. and persisted until 5:00 p.m., on the first day of flowering (Fig. 2: A-B). Flowers are off-white, petal length approximate 4 cm, and the outer whorl of petals gradually opens to a position that is nearly horizontal, thereby exposing the apertures of the pollination chamber. The apertures are approximately 5 mm in length and 3 mm in width (Fig. 1D, E). Additionally, a fruity-sweet fragrance can be perceived. The stigma is tender and succulent, with a thin layer of secretion appearing on the surface. The yellow stamens were tightly packed together and the anthers did not dehisce (Fig1. F). Toward the end of the stage, the outer whorl of petals converged inward, the pollination aperture closed, and the floral scent fades and difficult to smell.
- Stage V: interim phase (ca. 16h). The interim phase commences at approximately 5:00 p.m. on the first day of flowering and terminates at 9:00 a.m. on the second day of flowering (Fig. 2: B-C). Flowers are off-white and at this stage the outer whorl of petals pendulous, the pollination chamber is closed (Fig. 1G, H). The stigma is soft and easily dislodged, the stigma surface no longer produces secretion, and there is no perceptible scent ((Fig. 1 I).
- Stage VI: staminate phase (ca. 2h). The staminate stage commences at 9:00 a.m. on the second day of anthesis and persists until approximately 11:00 a.m. (Fig. 2: C-D). Flowers are off-white, pollination chambers remain closed, petal bases become deepen and petal tips soften, gaps between stamens widen begin to abscise, and the thecae dehisce pollen disperses inside pollination chambers (Fig. 1J, K). Stigma tip turns dark brown to black and abscise from ovary. At the end of this stage, the stamens and pollen are gradually fall from the receptacle along with the petals, within a very brief period (Fig. 1L).

### Petals fluorescence of *A. hainanensis*

**Fig. 3:**
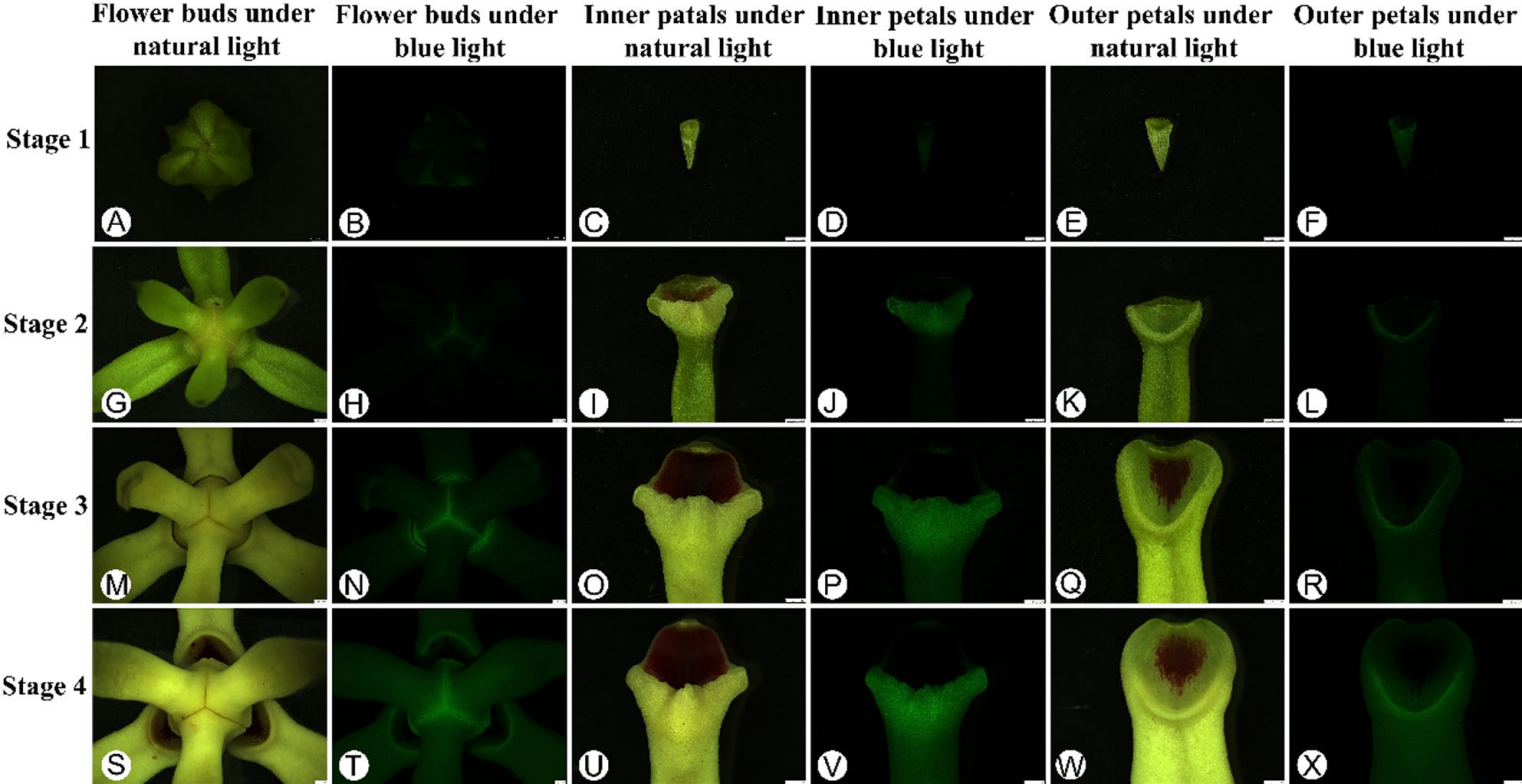
Autofluorescence phenomenon of *Artabotrys hainanensis.***A**, **G**, **M**, **S**: The flower bud under natural light; **C**, **I**, **O**, **U**: The inner petals under natural light; **E**, **K**, **Q**, W: The outer petals under natural light; **B**, **D**, **F**: The flower bud (**B**), inner petals (**D**) and outer petals (**F**) almost no fluorescence in stage 1; **H**, **J**, **L**: In stage 2, faint fluorescence appearing at the base of the pollination chamber (**H**), i.e., the raised areas of both the inner (**J**) and outer petals (**L**); **N**, **P**, **R**: In stage 3, the fluorescence at the base of the pollination chamber is distinct (**N**), and the fluorescence on the raised areas of both the inner (**P**) and outer petals (**R**) is also relatively noticeable; **T**, **V**, **X**: In the stage 4, the fluorescence at the base of the pollination chamber is extremely distinct (**T**), and the fluorescence on the raised areas of both the inner (**V**) and outer petals (**X**) is also very intense, showing a clear distinction from other parts. Scale bars:5 mm

**Fig. 4:**
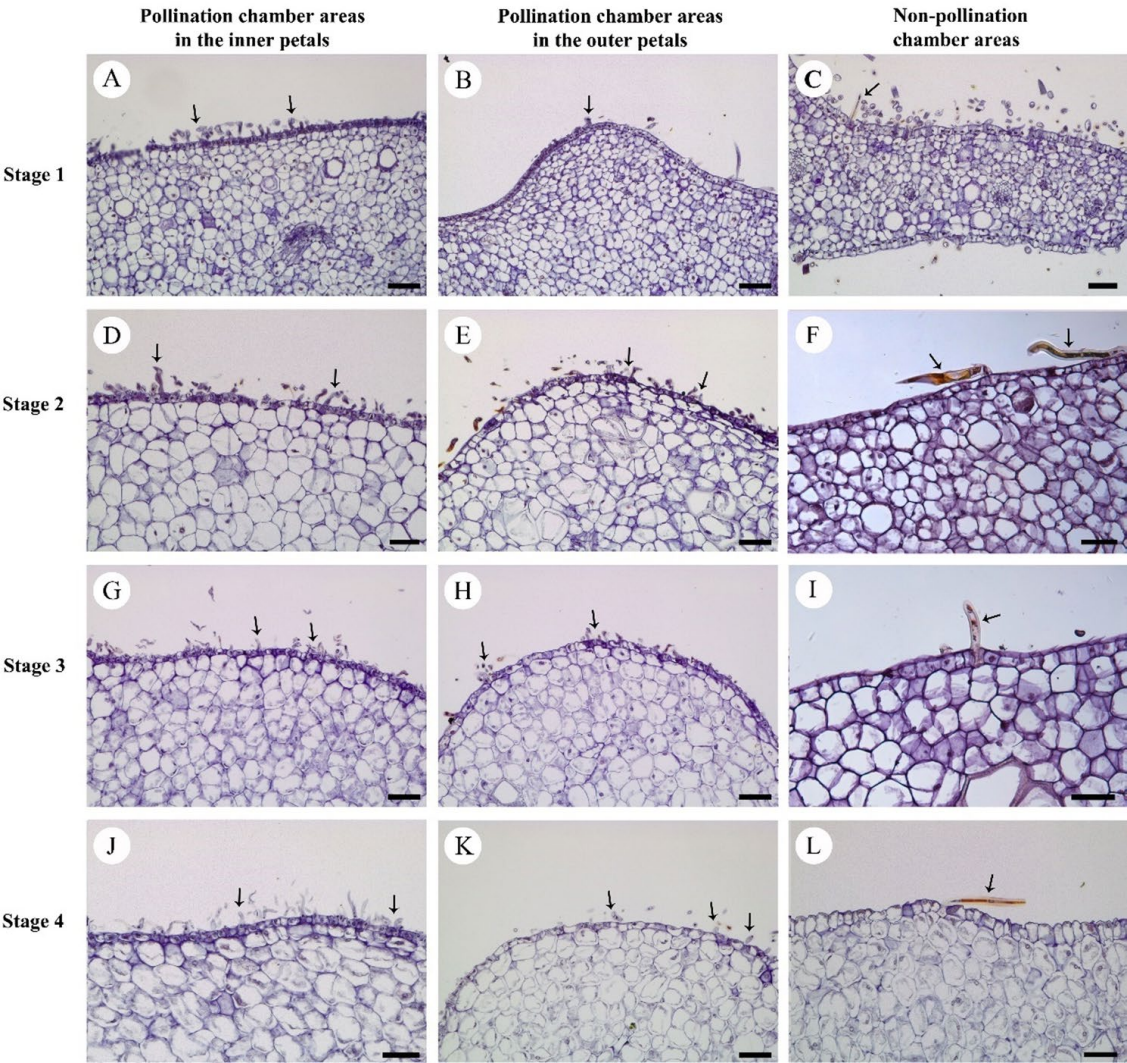
The structure of epidermal hair at different developing stages in *Artabotrys hainanensis*. **A**, **D**, **G**, **J**: With the flower development, the trichomes (arrows) in the base of the pollination chamber areas of inner petals progressively elongated and increased in density; **B**, **E**, **H**, **K**: The trichomes (arrows) in the base of the pollination chamber areas of outer petals become more elongated and denser; **C**, **F**, **I**, **L**: The trichomes are acicular erect and become sparser at the non-pollination chamber areas with the flower development. Scale bars: 50 μm

The pollination chamber was observed to emit green fluorescence under blue light illumination gradually increases with the petal development (Figs. 3 and 4). The fluorescence intensity was very faint with approximately 3.1 as measured by the ImageJ [25] (Fig. S1), but was almost indistinguishable by naked eye (Fig. 3A-F) at stage 1 (the flower bud is ca. 0.5 cm in diameter). The fluorescence intensity could be observed with an intensity of 11.6 (Fig. 3G-L, Fig. S1) when the petal developed to stage 2 (the flower bud is ca. 1.5 cm in diameter) and that increased to 20.9 after the pollination chamber formed (stage 3, the flower bud is ca. 3 cm in diameter) (Fig. 3M-R, Fig. S1). The fluorescence intensity of the pollination chamber was 24.3 at stage 4 (the flower bud is ca. 4 cm in diameter) (Fig. S1), and the outer whorl of petals opens to expose the pollination chamber apertures, which shown as three bright circles around the center of the flower (Fig. 3S-X). In addition, the fluorescence of non-pollination chamber areas was always inconspicuous, and the fluorescence intensity of the pollination chamber in the inner petals was consistently brighter than that of the outer petals.

### The trichomes morphology

The petals transverse sections at the pollination chamber areas and non-pollination chamber areas of the two whorls petals show that except the trichomes exhibit various morphology, no significant differences were observed in histological structures between the inner and outer petals (Fig. 4). The trichomes were curly and dense at the pollination chamber areas (Fig. 4A, B, D, E, G, H, J, K), whereas they were acicular erect and sparse at the non-pollination chamber areas (Fig. 4C, F, I, L).

**Fig. 5:**
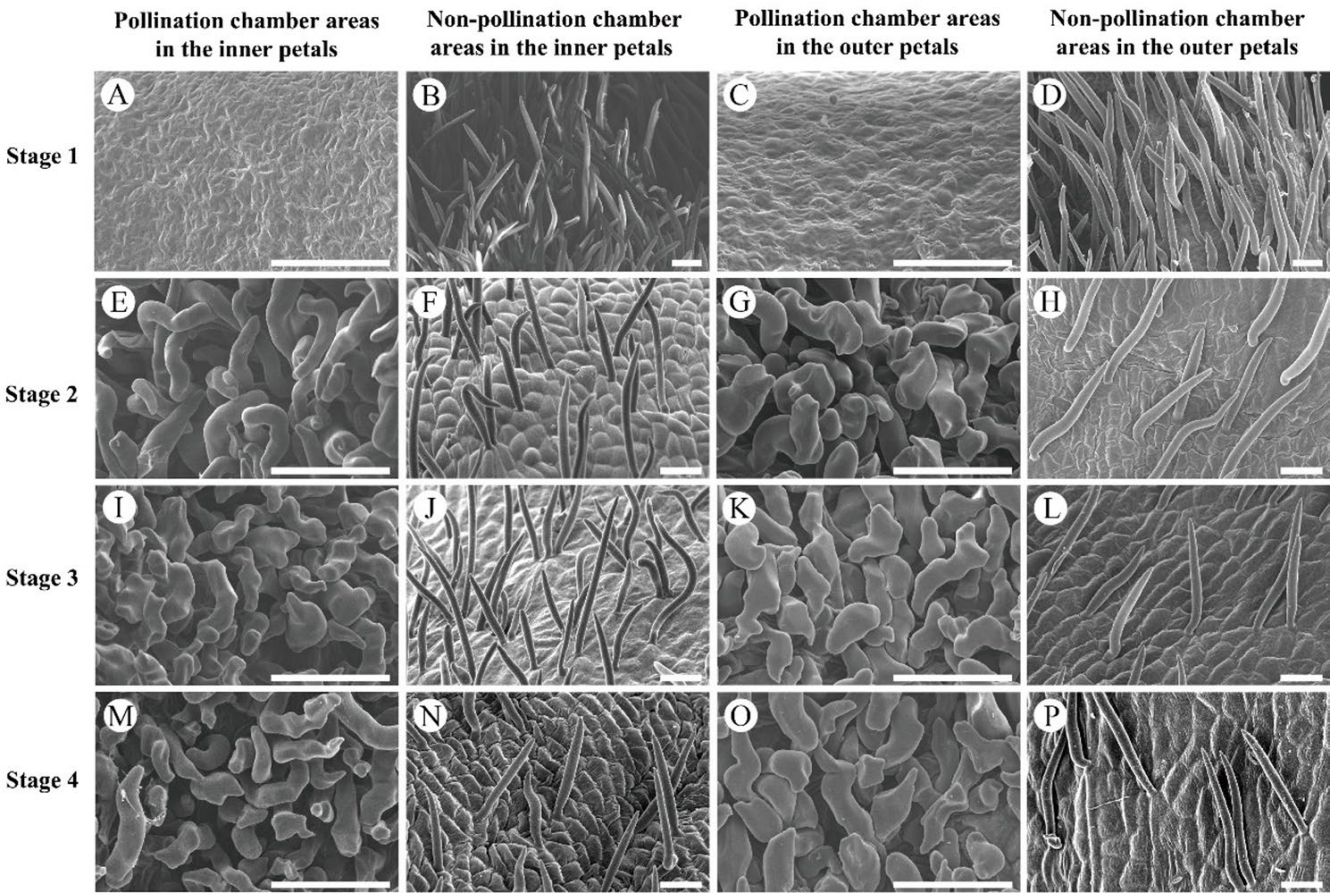
The morphology of epidermal hair at different developing stages in *Artabotrys hainanensis*. **A**-**D**: At stage 1, the pollination chamber areas are smooth and without trichomes (**A**, **C**) and non-pollination chamber are densely covered with erect acicular trichomes (**B**, **D**); **E**-**H**: At stage 2, trichomes at the pollination chamber areas are curled and twisted (**E**, **G**) and erect acicular trichomes at non-pollination chamber areas relatively reduced (**F**, **H**); **I**-**L**: At stage 3, the density of trichomes increased, with their morphology becoming further curved and entangled (**I**, **K**), while erect acicular trichomes at non-pollination chamber areas further reduced (**J**, **L**); **M**-**P**: At stage 4, trichomes at the pollination chamber areas further enlarged (**M**, **O**), while erect acicular trichomes at non-pollination chamber areas further reduced (**N**, **P**). Scale bars: 50 μm

The morphology of petal trichomes was observed using a scanning electron microscope, the pollination chamber areas were smooth and free of trichomes at stage 1(Fig. 5: A, C), whereas the non-pollination chamber areas were densely covered with erect acicular trichomes (Fig. 5: B, D). The densely curled and twisted trichomes were observed in the pollination chamber areas at stage 2 (Fig. 5: E, G), and the density of trichomes in non-pollination areas relatively reduced and exhibited an erect acicular morphology resemble that at stage 1 (Fig. 5: F, H). Trichomes in the pollination chamber further curled and twisted and increased in length as the petals grow to stage 3 and stage 4 (Fig. 5: I, K, M, O), and as the petal elongated, the distribution of trichomes in non-pollination chamber areas became more sparser (Fig. 5: J, L, N, P). The averages length of trichomes in the pollination chamber areas of mature petal was 50 μm, and the density of trichomes per unit area was about 2000 hairs/mm^2^, while the average length of trichomes in the non-pollination chamber was 700 μm, and the density of trichomes per unit area was about 35 hairs/mm^2^.

### Fluorescent compound lignin and content

**Fig. 6:**
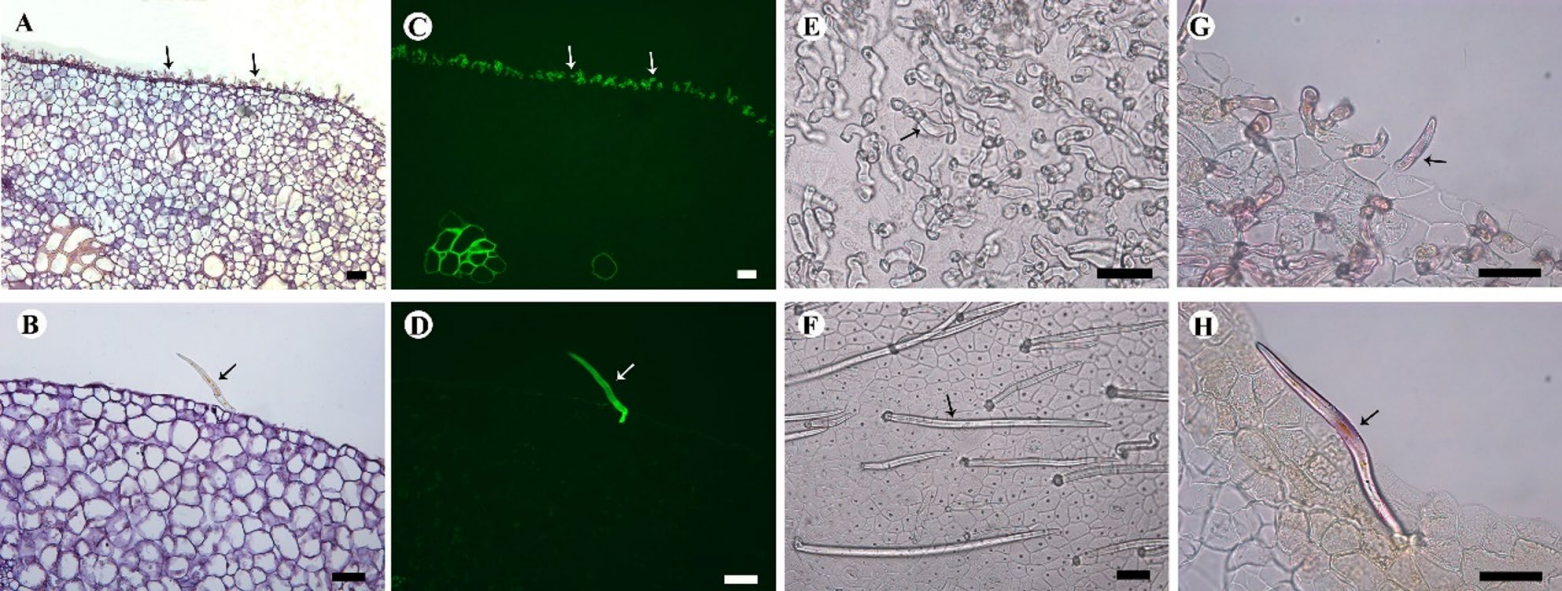
Structure and composition of trichomes in *Artabotrys hainanensis*. **A**: Paraffin section of the raised part of the pollination chamber, showing the trichomes (arrows); **B**: Paraffin section of non-pollinating chamber, showing the trichomes (arrow); **C**: Trichomes (arrows) of the raised part of the pollination chamber emit green fluorescence when excited by blue light; **D**: Trichomes (arrow) of non-pollination chamber emit green fluorescence when excited by blue light; **E**: Epidermal hairs (arrows) of the pollination chamber area; **F**: Trichomes (arrow) of non-pollination chamber region; **G**: Trichomes of the pollination chamber area were stained red (arrows) after phloroglucinol staining; **H**: Trichomes of the non-pollination chamber region were stained red (arrow) after phloroglucinol staining. Scale bars: 50 μm

Transverse paraffin sections of petals emitted bright green fluorescence of trichomes and vascular bundles under the blue light of a fluorescent stereomicroscope (Fig. 6A-D). The fluorescent properties of trichomes are like those of the vascular bundles, and the latter can fluoresce due to the presence of the fluorescent compound lignin, so it is assumed that the petals fluorescence of *A. hainanensis* flowers may be derived from the lignin within the trichomes. To prove the above hypothesis, the epidermal hair together with the epidermis cell layers of the mature petals were separated from petal tissue (Fig. 6E, F). Then stained with phloroglucinol, trichomes of both morphologies were stained red, while the epidermal cell layer was not colored, indicating that both trichomes contained lignin and the epidermal cells lacked lignin (Fig. 6G, H).

The lignin content of the petals gradually increased as flowers developed from stage 1 to stage 4 (Fig. S2). The lignin content of the non-pollination chamber part of the petals was higher than that of the pollination chamber part of the petals only at the early bud stage (stage 1), and thereafter, the lignin in the pollination chamber areasgradually accumulated, and its lignin content was significantly higher than that of the non-pollination chamber areas (stage 2-stage 4). Corresponding to the lignin content, the fluorescence intensity of the petal pollination chamber areas also gradually increased with flower development. The fluorescence of the pollination chamber area was more obvious than that of the non-pollination chamber areas, and the fluorescence of the pollination chamber areas in the inner whorl petals was brighter than that of the outer whorl petals. That is, the lignin contained within the trichomes determines the fluorescence intensity emitted by the petals of *A. hainanensis* flower, while the number and density of the trichomes affects the lignin content in the petal.

### Floral visitors and pollinator flower visiting behavior

**Fig. 7:**
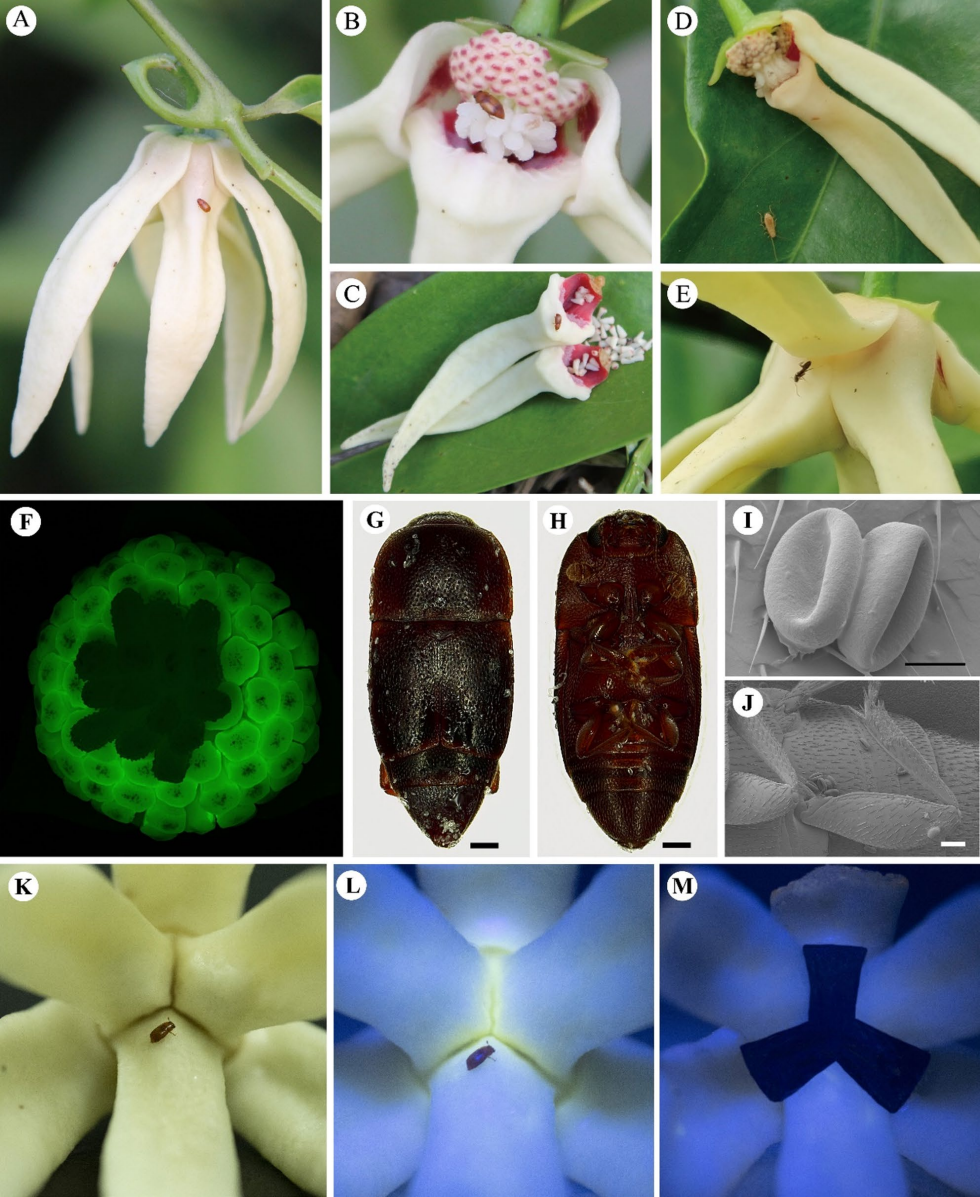
A survey of pollinators of *Artabotrys hainanensis*. **A**: Pistillate phase beetle began to visit flower; **B**: Beetle move in chamber; **C**: Bettle release at the end of the staminate phase; **D**: The larvae of Blattaria visit flower; **E**: Ant visit flower; **F**: The autofluorescence of anther under blue light; **G**, **H**: Carpophilinae beetle; **I**: Pollen of *Artabotrys hainanensis*; **J**: Carpophilinae beetles with pollen on their bodies; **K**: Beetles visit flowers in natural light; **L**: Under blue light, the autofluorescence of the base of the pollination chamber attract beetle to visit flowers; **M**: Under blue light, the base of the pollination chamber is covered with black paint. Scale bars: G, H = 250 μm; I = 20 μm; J = 100 μm

The insect observed in the field identified three kinds visiting *A. hainanensis* flowers during the anthesis period, including small beetles (Fig. 7A-C), cockroach larvae (Fig. 7D), and ants (Fig. 7E). The observation of cockroach larvae entering the pollination chambers was noted to occur during the pistillate phase, with the larvae remaining inside for approximately 30 min before climbing out, when the pollination chamber was opened by hand, some petals and stamens were blackened by eating (Fig. 7D). Only one visit of cockroach larvae to flowers was observed and they did not move from flower to flower, which assumed that they only feed on petals and stamens and therefore cockroach is not considered an effective pollinator, but an opportunist. The ants mainly crawled on the petals and moved around the base of the petals (Fig. 7E), were not observed entering the pollination chamber, and the limited mobility of the ants led to consider that the ants were not effective pollinators of *A. hainanensis*.

The small beetles were observed to fly into the petals of flowers in the pistillate phase, subsequently crawling towards the basal part of the flower and entering the pollination chamber through the apertures. Gently open the petals of a flower that had small beetles entered, it was found that the small beetles mostly stayed near the stigma and touching the stamens as they moved in the flower (Fig. 7B), and the anther emitted green fluorescence under blue light (Fig. 7F). After entering the pollination chamber, most of the small beetles stayed inside, and at night when the apertures of the pollination chamber closed the beetles were trapped inside until the end of the staminate phase when the petals fell off and the beetles were released (Fig. 7C). In field observations, beetles were leaving a flower at the end of the staminate phase and flying to visit another *A. hainanensis* flower at pistillate phase. The small beetles (ca. 3 mm long, abdomen ca. 2 mm wide) were captured back to the laboratory and identified as belonging to the subfamily Carpophilinae (Coleoptera: Nitidulidae) via stereomicroscope and scanning electron microscopy. The beetles carried pollen from *A. hainanensis* flowers (Fig. 7G-J), combined with the observation that the timing of the beetles’ activities corresponded to the flower anthesis, suggests that the small beetles of the subfamily Carpophilinae are effective pollinators.

A paired samples t-test was performed to examine the difference in pollinator behaviors before and after artificially blackened petal pollination chamber. The results revealed that after blocking the pollination chamber of the flower petals which could emit fluorescence, both the visiting frequency and staying time of the pollinators on flowers were significantly reduced (*t* (4) = 4.447, *p* = 0.011; *t* (4) = 6.683, *p* = 0.003, respectively), and the beetles needed to spend longer time to find the flower (*t* (4) = -6.52, *p* = 0.003) (Fig. 7K-M; Table 1). Besides, beetles in glass vials of group A visited flowers are disoriented, crawling in all directions of the petals, indicating that pollinators were unable to accurately locate at the flower when the fluorescence of petals was blocked.

**Table 1:** Records of laboratory insect visits to flowers

	Autofluorescence uncovered	Autofluorescence covered
Average staying time (minutes)	126 ± 17.49a	15 ± 1.7b
Average visiting frequency (times/hour)	3 ± 0.54a	1 ± 0.32b
Mean time from bottle entry to flower alighting for a beetle (minutes)	6 ± 0.58b	15 ± 1.63a

Values are means ± SE, *n* = 5

## Discussion

### The pollination chambers as pollinator traps increase pollination efficiency

The *A. hainanensis* flower exhibits hermaphroditic and protogyny characteristics, and possesses a close-type pollination chamber, which was considered as the pollinators trap, a structure that prevents a flower visitor from leaving before pollination has been completed [19]. Upon entering the pistillate phase, the pollination chamber apertures were opened, and remained opening throughout the pistillate phase, allowing pollinators access to the chamber. When the pistillate phase was concluded, the pollinator chamber apertures were closed, blocking the pollinators leaving from the chamber, and trapping the potential pollinators into the chamber. During the latter, due to the closed pollination chamber, the pollinators can only move around the pollen-filled pollination chamber, carrying enough pollen on their body. In field observations, the end of the staminate phase of one flower of *A. hainanensis* was found to coincide with the peak of stigma receptivity of another flower at pistillate phase, and the pollen-laden pollinators exited the flowers at the end of the staminate phase and were attracted by flowers in the pistillate phase, subsequently entering their pollination chamber and transferring pollen to the stigma. Alternatively, they may feed on the nectar-rich secretions within the flower and subsequently fly to another flower, also in the pistillate phase, with the potential to pollinate several flowers.

In most species of Annonaceae, such as *Goniothalamus tapisoides* [18], *Dasymaschalon trichophorum*, and, *Friesodielsia borneensis* [19], the pollination chamber acts as a pollinator trap, and exhibits resemble dynamic changes in anthesis as *A. hainanensis*, although the duration of the phases in these species may be different, but the anthesis phenology of these species was found to cooperate with the activity patterns of pollinating insects to ensure efficient pollination. Lau et al. [19] posits that such pollinator traps, when acted upon by pollinator chambers, possess distinct selective advantages. These include the expansion of the range of potential pollinators based on their activity patterns, the prolongation of the time during which stamen pollen is deposited on the bodies of pollinators, and the enhancement of inter-flower movement of pollinators. The ultimate purpose of this process was to successfully pollination and promote outcrossing and fruiting. Although the structure of pollination chambers and the operational mechanisms of pollinator traps may be different between species, this is a common feature in Annonaceae, and is likely to be a key evolutionary innovation of considerable functional importance [19, 26].

### Flower fluorescence attracts pollinators to visit flowers

The flower fluorescence is widespread in plants and has been proposed as a potential visual signal to attract pollinators [2, 4, 9]. Pollen and anthers of many plants usually emit bright blue fluorescence under UV light [5, 27], which may protect the genes in pollen from harmful UV energy through a transduction of absorbed UV to the fluorescence [28, 29], and may be the attraction of pollinators by highlighting pollen as food as well [9]. The pollen of *A. hainanensis* also exhibits pronounced fluorescence (Fig. S3), yet it could not be detected by the pollinators outside, even when pollen was dispersed during the staminate phase, since the close-type pollination confines the pollen effectively. Olfactory signals typically act over long distances, as volatile molecules disperse widely and can be detected from afar [30, 31]. In contrast, once pollinators are in close proximity to the flowers, visual signals become more important at short distances by indicating the precise location of rewards [32–34]. The diffuse nature of these olfactory signals makes it difficult for pollinators to precisely locate the flower using their sense of smell alone [30, 31], especially in some species with pollination chambers, such as Annonaceae, Myristicaceae, Araceae and Dipterocarpaceae [35–37]. I t would be with great effort for the pollinating insects to enter the pollination chamber through the narrow aperture in a relatively short time. Fortunately, the fluorescence exhibited by the two whorls petals provided an orientation signal for the position of the pollination chamber aperture. The effective pollinator of the *A. hainanensis* flower was the small beetles, which their eyes could perceive a broad spectrum of light, ranging from 340 nm (ultraviolet, UV) to 540 nm (green light) [38–40]. We suggested that the green fluorescence around the aperture of the pollination chamber could guide the pollinators through the apertures and enter the chamber with precision. It was verified when the fluorescence around the aperture of the pollination chamber was artificially obscured in the lab. We observed that the frequency of the beetles visiting the flowers was reduced. Even the beetles that landed on the flowers, they became disoriented and crawled randomly on the petals, unable to locate accurately the aperture of the pollination chamber and enter it. It indicated that the pollinators can be attracted to visit the flowers by the olfactory, but they cannot accurately localize the pollination chamber apertures without the fluorescence around the aperture. In *A. hainanensis*, the challenge of a closed pollination chamber is overcome by strong autofluorescence at the base of the petals, which provides visual guidance that directs beetles—initially attracted by long-range scent—from long-range olfactory attraction to pinpointing the chamber opening via short-range fluorescent cues, thus ensuring successful pollination.

### Lignin acts as a fluorescent component in the flower

Most flowers typically contain a diverse array of pigment compounds that can exhibit fluorescence in response to varying light irradiation [4, 7, 8, 41]. The compounds that make petals fluoresce of the flowers were pigment molecules, including betaxanthin (*Portulaca grandiflora* and *Mirabilis jalapa*) [7, 8], aurones (*Antirrhinum majus*) [41], and flavonoids (*Ornithogalum thyrsoides*) [4]. However, the substance that made the petals of *A. hainanensis* fluoresce was lignin, which is unique and noteworthy. The current research on lignin fluorescence characteristics has been no reports of lignin present in floral structures that have demonstrated its fluorescent properties with specific functions, but was primarily concerned with utilizing it for the cell structure and cell type of xylem and vascular bundles [10, 12–14].

In *A. hainanensis*, the fluorescence intensity of the petals is dependent on the lignin content within the trichomes, that exhibited both an erect morphology and a curly one, with the former sparsely distributed at the non-pollination chamber areas, and the later densely distributed at the pollination chamber areas, with higher lignin content and relative to the fluorescence of the non-pollination aperture. Lignin has a broad range of autofluorescence emission and can be excited with both UV and visible light, emitting blue, green and red fluorescence [12, 42–44]. The green fluorescence emitted by lignin under visible light could attract beetles (Nitidulidae), which have visual receptors that are sensitive to green light. The investigation of this present study revealed that lignin fluorescence can serve as an optical signal in the context of plant-animal interactions, particularly between insects and plants, analogous to other flower fluorochrome molecules. As a widespread component of plant organisms, the distribution of lignin in floral structures is a topic worthy of further investigation and the function of lignin fluorescence phenomena in flowers deserves attention.

## Conclusion

*Artabotrys hainanensis*, a species of Annonaceae, exhibits hermaphroditic and protogyny characteristics, and possesses a close-type pollination chamber, of which a narrow aperture was exposed in only 8 h during flowering. The flower exhibits green fluorescence at the base of the inner and outer whorls of petals, due to the lignin contained in the trichomes on the petals. The lignin emits green fluorescence at a wavelength of 500–650 nm under blue light, which could perceived by the pollinators (Nitidulidae), with visual receptors and sensitive to green light. The green fluorescence could guide the pollinators entering the pollination chamber through the narrow aperture in the short exposing time, and ensuring successful pollination.

## Data Availability

All the data are contained within the article.
